# Colonic Interposition between the Liver and Diaphragm: “The Chilaiditi Sign”

**DOI:** 10.1155/2016/2174704

**Published:** 2016-03-08

**Authors:** Nita Nair, Zeina Takieddine, Hassan Tariq

**Affiliations:** Department of Medicine, Bronx-Lebanon Hospital Center, 1650 Selwyn Avenue, Suite No. 10C, Bronx, NY 10457, USA

## Abstract

A 90-year-old wheelchair bound male was brought to the emergency department with complaints of worsening abdominal pain over the last 2-3 days. The patient also had difficulty in passing urine. Abdominal examination revealed tenderness in the umbilical and hypogastric area without rebound tenderness or guarding. Computed tomography (CT) of the abdomen showed a loop of colon interpositioned between the liver and the right hemidiaphragm (the Chilaiditi sign), mimicking free air. Foley's catheter was placed and the patient was managed conservatively. The patient clinically improved with improvement of the abdominal pain.

## 1. Case Presentation

A 90-year-old wheelchair bound male was brought to the emergency department with complaints of worsening abdominal pain over the last 2-3 days. The pain was 7/10 in intensity, gradual in onset, and nonradiating, with no exacerbating or relieving factors. The patient also had difficulty in passing urine. He denied nausea, vomiting, fever, or any change in the bowel habits. His medical history was significant for Parkinson's disease and glaucoma. He had no significant past surgical history. He denied tobacco, alcohol, or illicit drug use.

On examination, he was afebrile, with a blood pressure of 167/87 mm of Hg, pulse of 103/minute, respiration rate of 18/minute, and oxygen saturation of 95% on room air. Precordial examination revealed normal heart sounds without any murmur or gallops. Auscultation of lungs revealed bilateral air entry without any adventitious sounds. Neurological examination revealed pill-rolling tremor of upper extremities, rigidity, and increased tone with decreased strength of 4/5 in all four extremities. Abdominal examination revealed tenderness in the umbilical and hypogastric area without rebound tenderness or guarding.

Laboratory studies were significant for serum potassium of 5.1, blood urea nitrogen (BUN) of 55, and creatinine of 6.3.

**Figure 1:**
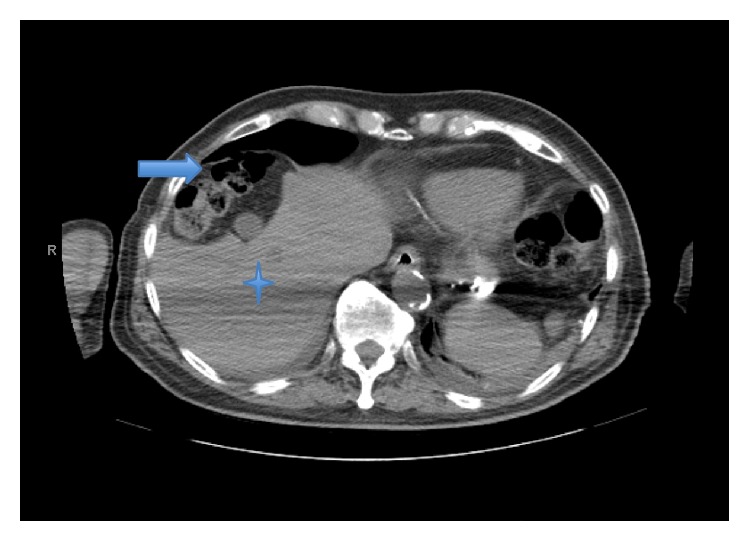
CT of the abdomen (axial section) showing a loop of colon (arrow) between the liver (asterisk) and the right hemidiaphragm.

**Figure 2:**
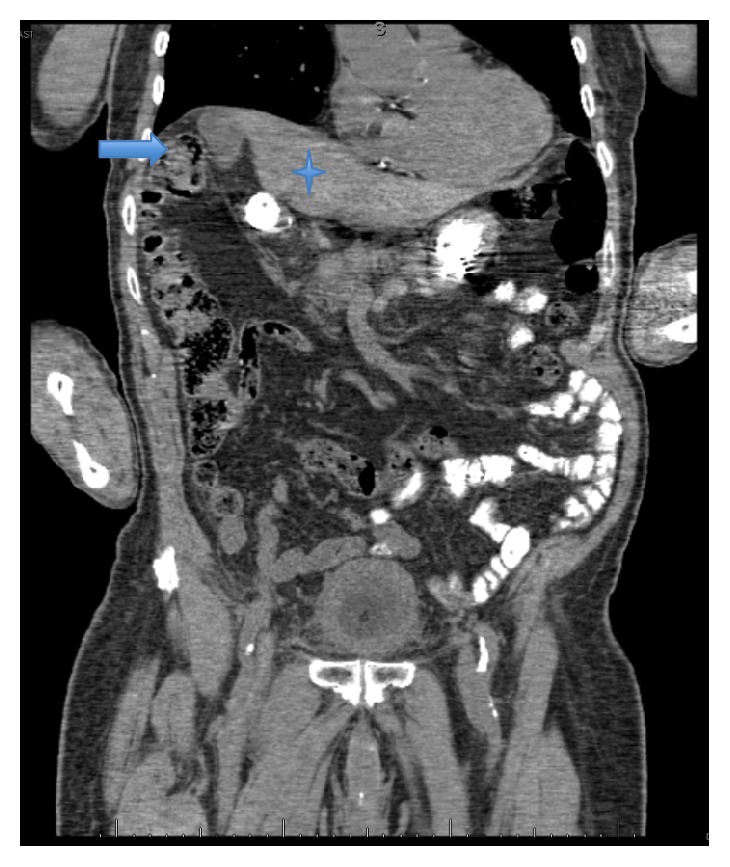
CT of the abdomen (coronal section) showing loops of colon (arrow) between the liver (asterisk) and the right hemidiaphragm.

Computed tomography (CT) of the abdomen showed a loop of colon interpositioned between the liver and the right hemidiaphragm, mimicking free air. Axial section (Figure 1) and coronal section (Figure 2) are shown. Also seen were bilateral perinephric stranding, right-sided hydronephrosis, and hydroureter with no obstructing calculus and thickening of the bladder wall.

Foley's catheter was placed and the patient was managed conservatively. The patient clinically improved with improvement of the abdominal pain.

## 2. Discussion

Chilaiditi sign, a segmental interposition of a loop of large intestine (usually hepatic flexure of colon) or small intestine (3–5% of cases) between the liver and diaphragm, is a radiographic finding named after the Greek radiologist Demetrius Chilaiditi from Vienna, who first described this sign in a small case series of 3 patients in 1910. This colonic interposition may be asymptomatic (Chilaiditi sign) or may be accompanied with broad spectrum of (gastrointestinal) symptoms (Chilaiditi syndrome) [1].

Chilaiditi sign has an incidence of 0.25% to 0.28% worldwide with a marked male predominance (male to female, 4 : 1). It can be either congenital or acquired. The predisposing factors of this condition may be congenital such as an elongated, redundant, and hypermobile colon, laxity, or absence of suspensory ligaments. Acquired causes may include a small liver due to atrophy in cirrhosis or hepatectomy, ascites, substantial weight loss in obese patients, and abnormally high diaphragm in conditions such as diaphragmatic muscular degeneration or phrenic nerve injury and rarely may occur due to excessive aerophagia [1, 2].

It is mostly diagnosed as an incidental finding on a chest X-ray or abdominal computed tomography (CT), which can be present temporarily or permanently [3]. On a chest X-ray it presents as air under the right hemidiaphragm and can be mistaken for pneumoperitoneum and can lead to unnecessary surgical intervention if not recognized correctly [3]. Interventions are not required for asymptomatic patients with Chilaiditi sign and the treatment is usually conservative.

In patients presenting with Chilaiditi syndrome, the most common symptoms are gastrointestinal such as abdominal pain, anorexia, nausea, vomiting, and constipation, followed by respiratory distress, and, less frequently, cardiac such as angina like chest pain [4]. Complications of Chilaiditi syndrome may include volvulus of the cecum, splenic flexure, or transverse colon. Cecal and rarely subdiaphragmatic perforated appendicitis has also been reported [4].

Initial management of Chilaiditi syndrome is conservative with bed rest, intravenous fluid, bowel decompression, enemas, and laxatives. If the patient does not respond to initial conservative management, and either the obstruction fails to resolve or there is evidence of bowel ischemia, then surgical intervention is indicated [4, 5].

In our patient the abdominal pain was secondary to obstructive uropathy with acute renal failure, which resolved with indwelling Foley's catheter placement and Chilaiditi sign was just an incidental finding.

Chilaiditi sign and Chilaiditi syndrome are rare entities and are therefore often misdiagnosed in clinical practice; however, they may be accompanied by a series of severe complications, such as bowel obstruction and perforation. They can lead to unnecessary surgical intervention if not recognized correctly.
